# Energy-efficient recovery of fermented butyric acid using octyl acetate extraction

**DOI:** 10.1186/s13068-022-02146-6

**Published:** 2022-05-06

**Authors:** Hyeon Woo Oh, Seong Chan Lee, Hee Chul Woo, Young Han Kim

**Affiliations:** Department of Chemical Engineering, Pukyong National University, 365 Shinsun-ro, Nam-gu, Busan, 48547 South Korea

**Keywords:** Butyric acid recovery, Solvent extraction, Energy-efficient, Biofuel

## Abstract

**Background:**

A butyric acid recovery process using octyl acetate is proposed, and the design details of the extraction and subsequent distillation processes were investigated. Ternary equilibrium data for the extractor design were derived from molecular simulations and experimental measurements.

**Results:**

A new procedure for estimating the thermodynamic parameters was introduced to determine the effect of the parameters on extractor design by comparison with previously reported parameters. Using the proposed recovery process with the newly estimated thermodynamic model, 99.8% butyric acid was recovered from the fermentation broth at a recovery rate of 99%. The energy demand for the proposed process was found to be lower than the average demand for several reported butyric acid recovery processes.

**Conclusions:**

The investment cost is projected to be lower than that of other butyric acid processes due to the high efficiency of extraction solvent. The recovery cost of butyric acid was comparable to its selling price.

**Supplementary Information:**

The online version contains supplementary material available at 10.1186/s13068-022-02146-6.

## Background

Butyric acid is a valuable biofuel that can offer an alternative to fossil fuels due to several advantages, such as its high carbon number and oxygen content, as well as its low-pollution characteristics and environmental sustainability of its sources [1]. It has also been used as an animal feed additive, both as a supplement and as an antibiotic, owing to its anti-pathogenic properties and regulations regarding the use of antibiotics in animal feed. Therefore, it is accepted as the most efficient energy source for animals, especially for poultry and swine [2]. Butyric acid is well known for its anticancer effects as it induces morphological and biochemical differentiation in a variety of cells. On a global scale, butyric acid production from dairy industry wastewater is projected at 5.39 Mt/year [2], and sewage sludge fermentation and valorization in urban wastewater treatment plants not only reduces landfill disposal, but also produces valuable butyric acid [3].

A common difficulty in the biofuel recovery process arises from its low content in the fermentation broth [4–6], which is generally less than 10% in water. Distillation and extraction are well-developed technologies employed for chemical product separation [7–11]: the former consumes a large amount of energy, and the latter requires the implementation of appropriate solvents. Considering product volatility and water content, bioethanol is the only viable fuel for which distillation has been adopted as the recovery process. While many studies have suggested extraction as a common recovery technology [2, 12–15], the recovery efficiency hinges on finding an adequate solvent. Although general guidelines for solvent selection have been introduced by a number of studies [16–18], the limited availability of thermodynamic data for the liquid–liquid equilibrium (LLE) discourages butyric acid recovery via extraction despite the numerous benefits of this approach, including low energy demand. Many previous studies on butyric acid recovery used co-extractants containing ion-pairing reagents such as trioctylphosphine oxide [19], trioctylamine [20], and ionic liquids [21]; however, dissolved salts could lower VFA recovery due to the co-extraction of salt anions and an increase in the pH of the aqueous solutions [2]. The extraction of an organic solvent without the co-extractant would provide a readily applicable process for butyric acid recovery [9], and its performance depends mainly on the selection of the solvent. In a previous study [9], the process was designed using the UNIFAC thermodynamic model, which could result in less accurate product recovery and purity. The application of experimental and molecular simulation data to the thermodynamic model provides a more accurate process simulation and design.

In this study, a new solvent for the recovery of butyric acid from the fermentation broth was proposed, and its extraction performance was evaluated by comparing with other hybrid extraction/distillation processes of butyric acid recovery. This study achieved the following: (i) octyl acetate as a single extractant was found to be the optimal solvent for butyric acid recovery; (ii) the thermodynamic data for the LLE were obtained from molecular simulations and experimental measurements, and their NRTL model parameters were estimated; and (iii) the energy demand and investment cost of the proposed process were compared with those of previously reported butyric acid recovery processes.

## Methods

### Solvent selection

Regarding acetic acid recovery from fermented broth, ethyl acetate exhibited the best performance among several common solvents [12], and octanoic acid diluted with toluene exhibited an excellent butyric acid recovery performance [22]. Hexanoic acid has been used for butyric acid recovery from fermentation broth [22], and octanoic acid has been extracted using methyl stearate in biodiesel [23]. Among the various C4 and C5 alcohols, 3-methyl-1-butanol enabled the highest recovery of butyric acid [24]. In the case of various volatile fatty acids (C2–C6), nonyl acetate has been used as the extraction solvent [9]. The above-mentioned findings of previous studies suggest that alcohols and esters are suitable solvents for the extraction of butyric acid from fermentation broth. Because the solvent is recycled after separation from the product by distillation, its boiling point has to be somewhat higher than that of butyric acid in addition to exhibiting a favorable extraction performance. The amount of solvent is considerably larger than that of butyric acid, and the solvent should preferably reside at the bottom during distillation to maximize energy efficiency. Most of the reboiler duty in the solvent recovery column is recovered by heat integration. Details regarding energy recovery are discussed in the section of Process‑design results. Among the high-boiling-point esters used as potential solvents for butyric acid extraction, octyl acetate was selected as the solvent for the proposed process based on its boiling point and extraction performance.

### Thermodynamic model

The commonly employed commercial design software Aspen Plus ver. 8.8 was utilized in this study to design the butyric acid recovery process. While the physical properties of the components in the proposed process were estimated using basic properties in the databases of the design program, many thermodynamic model parameters are not included in the databases. In particular, the model parameters for the LLE are rarely included in the databases. The NRTL model [25] was used to compute the vapor–liquid equilibrium (VLE) and LLE. The LLE model parameters are particularly important for achieving extraction computation accuracy [26]. In this study, the crucial NRTL model parameters for the LLE system of water/butyric acid/octyl acetate were derived from molecular simulation results and partially supported by experimentally obtained LLE results.

The UNIFAC model, which is widely used in the prediction of VLE, is not efficient for LLE prediction, and its parameters would produce an ineffective design in chemical process simulations. This is due to several limitations, such as the lack of experimental data with different functional groups under wide operational conditions and inadequate thermodynamic consistency tests for the experimental data [27, 28]. Meanwhile, the UNIQUAC model is commonly used for activity coefficient estimation such as the NRTL model comprising binary interaction parameters; moreover, the additional and adjustable parameter of non-randomness in the NRTL model provides a better prediction of equilibrium relations, as demonstrated in the example of a ternary system of water/butyric acid/cyclohexanone, similar to that of this study [29].

### Molecular simulation

Process design using software, such as Aspen Plus and PRO/II, begins with the selection of constituent components and estimation of their physical and thermodynamic properties. For many processes, the parameters of the thermodynamic model calculating the thermodynamic properties are not sufficiently provided by the software, which limits solvent searching for a prospective extraction process. A computer-aided solvent screening tool, the conductor-like screening model for real solvents (COSMO-RS) [30, 31], includes molecular simulations for eligible solvent selection.

Molecular simulation determines an optimal distribution of constituent molecules in a mixture for two different cells, either vapor and liquid cells, or two liquid cells. The distribution is determined by calculating the interacting potentials between inter- and intra-molecules. The minimum potential among molecules is obtained when equilibrium is reached between the cells. Practically, all atoms comprising the molecules are counted in the potential computation. An open program for molecular simulations, RASPA ver. 2.0.3 [32, 33] was used in this study. The molecular parameters for the simulation were implemented as transferable potentials for phase equilibria (TraPPE) parameters [34]. Details regarding the simulation are presented in [18, 35]. The computed distribution of molecules among the two cells was converted to equilibrium compositions between the two different phases.

### Experimental measurements

The equilibrium compositions of the LLE systems determined using molecular simulations were compared to those obtained experimentally. A ternary sample of the water/butyric acid/octyl acetate system was placed in a 40 mL vial and mixed using a magnetic stirrer for 2 h. The sample was allowed to settle overnight for phase separation. The composition of the separated phases was determined using high-performance liquid chromatography (HPLC, Model: HP-5890, Agilent). The instrument was equipped with a flame ionization detector (FID), and nitrogen was applied to the sample through an adsorption column. A capillary column (HP-FFAP) was used to separate the components of the sample. This procedure is described in [10].

### NRTL parameter estimation

An accurate measurement of LLE tie-line data does not lead to equivalent design results for an extraction process, unless an adequate set of NRTL parameters are provided to the process design software. One way to evaluate the soundness of the parameters is by generating a ternary diagram of the LLE system. Hence, designing an extraction process begins with this diagram [36–38].

When the parameters are estimated, the Gibbs free energy of mixing has to be at a minimum [39–41]; therefore, the estimation includes the following equation as a constraint in this study:

1 $$ {\text{min}}\quad \frac{{\Delta G_{{{\text{mix}}}} }}{RT} = \mathop \sum \limits_{k = 1}^{{N_{{\text{p}}} }} \,\mathop \sum \limits_{i = 1}^{{N_{{\text{c}}} }} l_{i}^{k} \left( {\ln x_{i}^{k} + \ln \gamma_{i}^{k} } \right) $$

where $\Delta G_{{{\text{mix}}}}$ is the Gibbs free energy of mixing, *x* is the mole fraction of component *i*, *l* is the component liquid fraction, and *R* and *T* are the gas constant and absolute temperature, respectively. *N*_p_ is the number of liquid phases, *N*_c_ is the number of components, and $\gamma$ is the activity coefficient computed using the NRTL model.

The conventional LLE parameter estimation approach, the *K*-value method, utilizes a phase equation [42] and iso-activity conditions while satisfying the material balance [43].

2 $$ x_{i}^{{\text{I}}} = {{z_{i} } \mathord{\left/ {\vphantom {{z_{i} } {\left\{ {1 + \left( {K_{i} - 1 } \right) L^{{{\text{II}}}} } \right\}}}} \right. \kern-\nulldelimiterspace} {\left\{ {1 + \left( {K_{i} - 1 } \right) L^{{{\text{II}}}} } \right\}}} $$

3 $$ x_{i}^{{{\text{II}}}} = K_{i} x_{i}^{{\text{I}}} $$

4 $$ K_{i} = \gamma_{i}^{{\text{I}}} / \gamma_{i}^{{{\text{II}}}} $$

where *z*_i_ is the feed composition, *K*_i_ is the equilibrium constant, and *L*^II^ is the liquid II fraction. As there are two unknown variables, namely the feed composition and liquid fraction in Eq. (2), the phase equation has to be solved iteratively [44].

Setting the *z*_1_ a value between the two experimentally obtained values of *x*_1_^I^ and *x*_1_^II^, rest of feed compositions are non-iteratively calculated using a trigonometric relation because the feed and two liquid compositions are on a line using the following equation:

5 $$ \nabla \cdot \left( {{\varvec{z}} - {\varvec{x}}^{{{\text{II}}}} } \right) = \nabla \cdot \left( {{\varvec{x}}^{{\text{I}}} - {\varvec{x}}^{{{\text{II}}}} } \right) $$

With a given feed composition of one component, the compositions of the remaining components can be calculated using Eq. (5). Furthermore, the liquid II fraction is determined using two Euclidean distances and the lever rule.

6 $$ L^{{{\text{II}}}} = {{d \left( {{\varvec{x}}^{{\text{I}}} , {\varvec{z}} } \right) } \mathord{\left/ {\vphantom {{d \left( {{\varvec{x}}^{{\text{I}}} , {\varvec{z}} } \right) } {d \left( {{\varvec{x}}^{{\text{I}}} , {\varvec{x}}^{{{\text{II}}}} } \right) }}} \right. \kern-\nulldelimiterspace} {d \left( {{\varvec{x}}^{{\text{I}}} , {\varvec{x}}^{{{\text{II}}}} } \right) }} $$

For an *N*_c_ component system, its sum of component fractions is:

7 $$ \mathop \sum \limits_{i = 1}^{{N_{{\text{c}}} }} z_{i} = 1 $$

In a ternary system, Eqs. (5) and (6) are solved as a two-dimensional problem. These relations eliminate the need for iteration to solve the phase equation, thereby simplifying parameter estimation in the conventional *K*-value method. Determining the binary interaction parameters in the NRTL model that result in the minimum deviation between the measured tie-line compositions and the computed values obtained from the phase equation using the *K*-value method constitutes the parameter estimation procedure. The proposed procedure simplifies parameter estimation by eliminating the iterative procedure in solving the phase equation, flash calculation.

### General process description

**Fig. 1:**
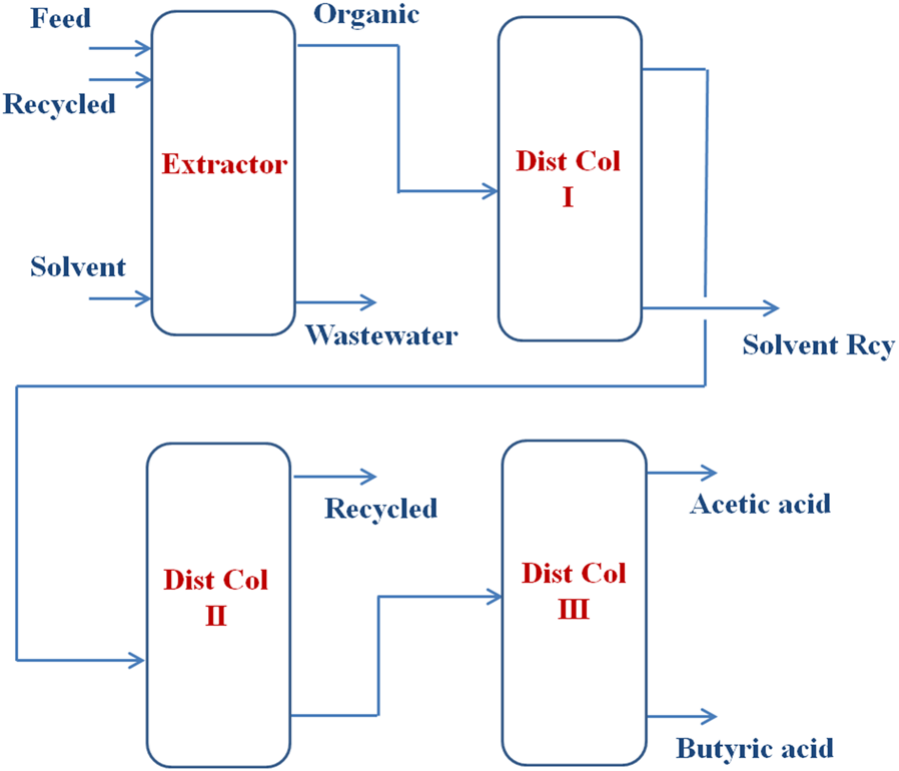
Brief schematic of the proposed butyric acid recovery process

The proposed process consists of an extractor and three distillation columns, as shown in Fig. 1. Because the amount of water in the extract after the extraction process was too large to be removed as an impurity of the butyric acid product, a recycled stream was included in the distillation products.

### Techno-economic analysis

The feed composition used for the proposed butyric acid recovery process was taken from [45], wherein butyric acid was produced from cane molasses using immobilized *Clostridium tyrobutyricum* via fed-batch fermentation at a pH of 6. The feed contained 2.67% butyric acid and 0.42% acetic acid in water. A lower pH is desirable for producing free acids that are easier to separate from the fermentation broth [45–47]. In another extraction process, 10% Alamine 336 in oleyl alcohol was used as the extractant, and all the butyric acid was extracted at an initial pH of 4.5 [46]. The NRTL parameters used in the process simulation executed by Aspen Plus ver. 8.8 are listed in Additional file 1: Table S1, and the derivation is explained in the following paragraph.

The extractor was designed for a 0.99 target recovery of butyric acid, and distillation columns were arranged to achieve a product purity of 99.5%. The columns were optimized for the minimum total annualized cost, comprising the investment and operating costs. The estimated cost equations were adopted from [48–51], and an index of 1638.2 (the Marshall and Swift equipment cost index in 2018) was applied. The payout time of the investment was 5 years, and 8000 h of operation per year was assumed. For heat integration, the distillation columns were preheated to the feed stage temperature using the recovered heat from the high-temperature streams. Pinch technology [52] was implemented to evaluate the availability of the recovered heat.

## Results and discussion

### NRTL parameters

**Fig. 2:**
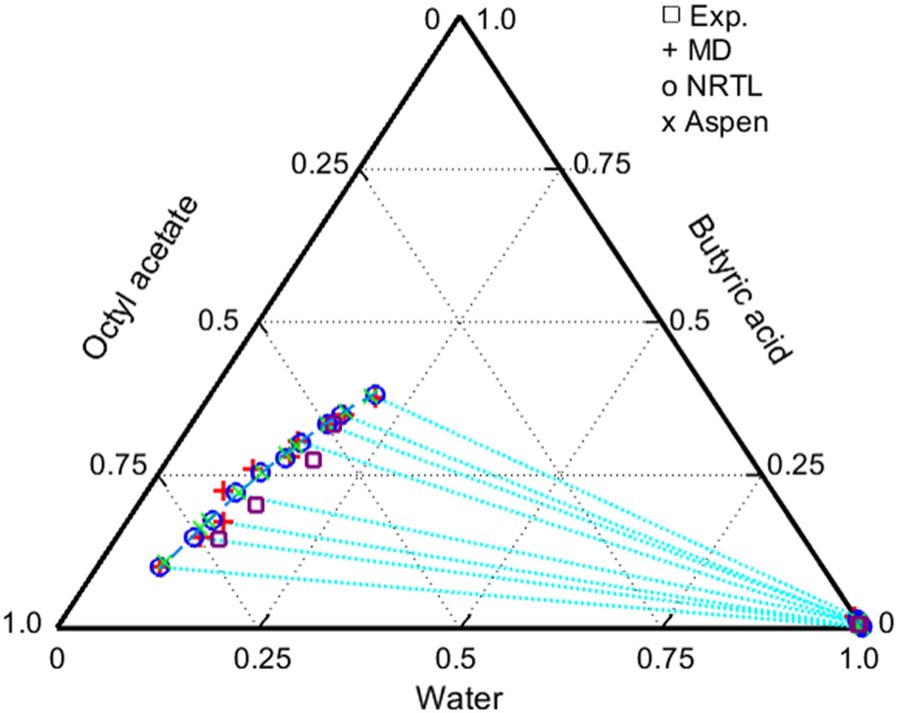
Ternary diagram of the water/butyric acid/octyl acetate system. Plus (+) symbols indicate results obtained via molecular simulation, squares represent the experimental values, the circles were computed using the NRTL model with the estimated parameters, and multiplication symbols were Aspen Plus computation with the parameters

Because experimental data for a ternary LLE system comprising water/butyric acid/octyl acetate were not available, a molecular simulation was conducted to determine the tie-line data for the system, and the binary interaction parameters were estimated using the procedure explained above. Furthermore, the tie-line data were measured as listed in Additional file 1: Table S2 to evaluate the accuracy of the estimated data obtained from the molecular simulation, as listed in Additional file 1: Table S3. Additional file 1: Table S2 includes the distribution coefficient and the solute selectivity. Figure 2 depicts the tie-line data of the molecular simulation and that obtained experimentally as plus symbols and squares, respectively. The left-hand symbols represent the organic phase compositions, and the right-hand ones do the aqueous compositions. Although there is some discrepancy between the two values, the results are well formulated in the NRTL model. The circles represent data computed with the model matched to the Aspen Plus calculation, denoted by multiplication symbols. The NRTL parameters were estimated using the molecular simulation and experimental results, and the obtained parameters were utilized by the Aspen Plus simulation for the process design. Note that the actual extractor design is based on the parameters listed in Additional file 1: Table S1 and obtained via Aspen Plus computation. The VLE parameters of the water/butyric acid and acetic acid/butyric acid binary pairs are listed in Aspen Plus, and the remaining binary systems were estimated from the VLE data obtained from the molecular simulation.

### Process-design results

**Fig. 3:**
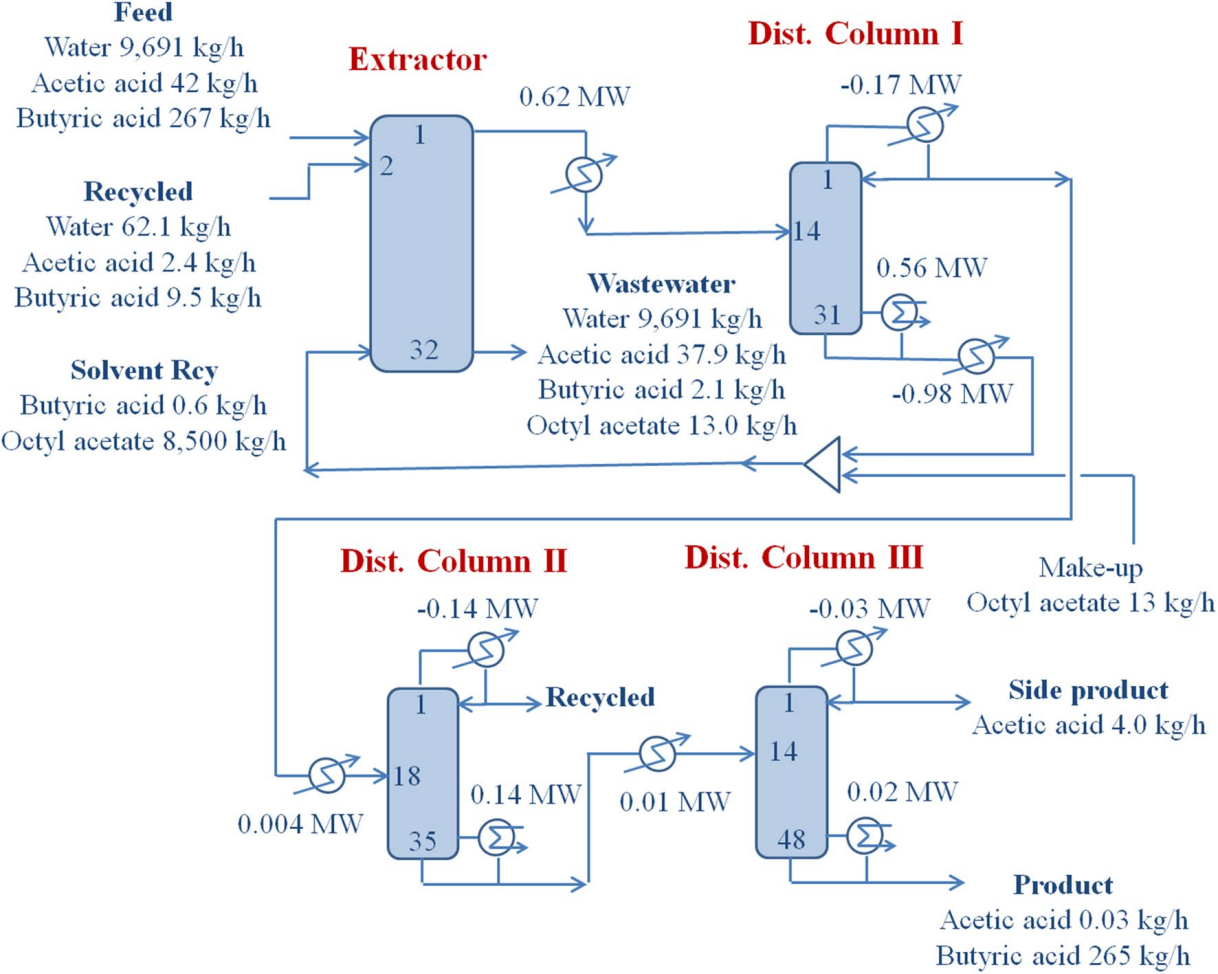
Process flow diagram of the proposed butyric acid recovery process

**Fig. 4:**
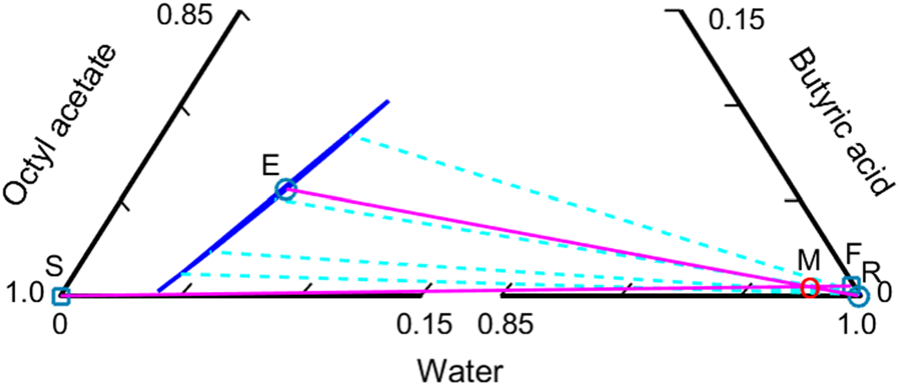
Expanded ternary diagram of streams and LLE tie-lines at the extractor. The blue line and light blue dashed-line denote the LLE tie-line data originally shown in Fig. 2. The F and S squares represent feed and solvent compositions, and circles E and R denote extract and raffinate compositions, respectively. M indicates the mixture composition of feed and solvent. Distances among the streams differ from the material balances obtained via the lever rule due to the discontinuous scale of the illustration

The design results regarding the equipment size, operational stream flow details, and heat transfer rate are shown in Fig. 3. More detailed information on equipment structure and operating conditions is provided in Table 1. Extractor performance is illustrated in Fig. 4, which is an expansion of the bottom portion of Fig. 2. The two squares denoted by F and S are the feed including the recycled stream and solvent compositions in mole fractions. The molar flow rates and compositions of the inlet and outlet streams at the extractor are summarized in Table 2. The expanded ternary diagram illustrates important information about the extractor. The proposed process of extractive water removal from the fermentation broth has the objective of high energy efficiency. Butyric acid is produced via an eco-friendly fermentation process using sustainable feed, which satisfies the goal of eliminating carbon dioxide emissions. The energy efficiency of the recovery process needs to be maximized by minimizing the energy consumption during distillation. Intrinsically, fermentation requires large amounts of water for microorganism operation. The association among water molecules necessitates a high energy input to achieve vaporization, the key process of distillation, and the number of vaporizations occurring repeatedly are in accordance with the number of stages in the distillation process. Reducing the distillation energy demand by including extraction is advantageous for maintaining the sustainability of butyric acid production via fermentation.

**Table 1:** Structural information and operating conditions used in the proposed process. The tray numbers are counted from the top

Variable	Extractor	Dist. Col. I	Dist. Col. II	Dist. Col. III
Structural				
Tray No.	32	31	35	48
Feed	1	14	18	14
Solvent	32			
Recycle	2			
Operating				
Pressure (MPa)-top	0.1	0.1	0.1	0.1
Temperature (°C)				
Overhead	25	141.8	100.0	130.1
Bottom	25	214.0	164.7	162.7
Feed (kg/h)	10,000	8831	343	269
Solvent (kg/h)	8501			
Make-up (kg/h)	13.0			
Recycle (kg/h)	74.0			
Product (kg/h)				
Overhead	8831	343	74	4.0
Bottom	9744	8488	269	265
Reflux (kg/h)	–	789	259	296
Vap. boil up(kg/h)	–	7491	1284	188
Cooling duty (MW)	–	− 0.17	− 0.14	− 0.03
Reboiler duty (MW)	–	0.56	0.14	0.02
Preheat/cool (MW)	− 0.98	0.62	0.004	0.01
Comp. (mass frac.)				
Feed				
Butyric acid	0.0267	0.0311	0.8001	0.9849
Product	Ovhd	Ovhd	Btm	Btm
Butyric acid	0.0311	0.8001	0.9849	0.9977

**Table 2:** Flow rates and stream composition at the extractor

Stream	Feed	Solvent	Extract	Wastewater
Composition				
Water	0.9929	0	0.0616	0.9986
Acetic acid	0.0014	0	0.0019	0.0012
Butyric acid	0.0058	0.0001	0.0558	0
Octyl acetate	0	0.9999	0.8807	0.0001
Flow rates (kmol/h)	545.3	49.3	55.9	538.7

A careful examination of the extraction process is necessary to ensure that the proposed process is energy efficient. The two principles of equilibrium and material balance are presented in Fig. 4. The close proximity of the connection line (pink) of the extract (E) and raffinate (R) compositions, and the tie-line of the organic and aqueous phases (light blue dashed), indicates that the equilibrium is correctly represented. The material balance between the feed and solvent is given as the ratio between the distance of square S and circle M, and that of circle M and square F, obtained using the lever rule. Circle M indicates the composition of the mixture of the two, which is not the actual value. Note that the diagram has a discontinuous scale for a magnified illustration of the components. The high water content obscures other component representations when a normal ternary diagram, such as that shown in Fig. 2, is used for the illustration. Likewise, the material balance between the extract and raffinate is indicated by the distances between the E, M, and R circles. Circle M is shared for the two material balances.

**Fig. 5:**
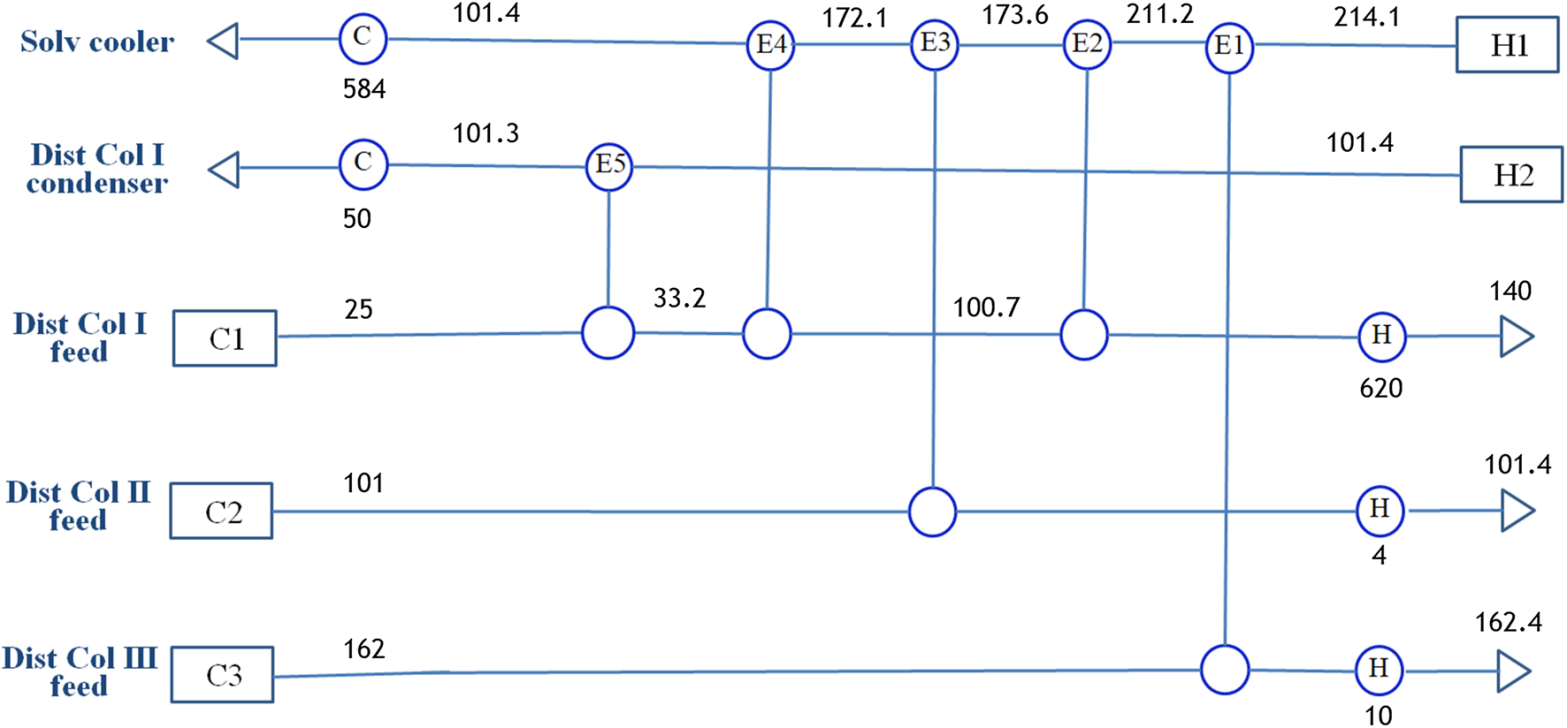
A diagram of heat exchanger network for heat recovery. Numbers below the circles represent the heat transfer rate in kW, and the numbers on the lines indicate the temperature in centigrade

The product-containing extract was purified by removing the recycled solvent, the recycled feed stream containing largely water, and the acetic acid side product, employing three consecutive distillation columns. The purified product comprised 99.8% butyric acid with a recovery rate of 99%. The distillation columns are fitted with pre-heaters for feed heating with heat recovered from recycled solvent cooling and the overhead condenser of the first distillation column. The heat recovery availability was examined using pinch analysis [52]. Figure 5 shows the heat exchanger network of 5 units with four input and output stream temperatures at each unit. The hot and cold composite curves indicate that sufficient heat supply is available.

### Economic evaluation

The economic evaluation of the proposed process is presented in Table 3. The investment cost includes column construction and heat exchanger fabrication, and the utility costs entail steam generation and cooling water supply. When a payout time of 5 years is considered, 1 kg of butyric acid recovery costs $0.23. The cost is comparable to the reported recovery cost of $0.53 [9] and a mixed VFA selling price of $2.70/kg [13]. As mentioned above, the energy efficiency of the proposed product recovery process is particularly important for a sustainable energy source.

**Table 3:** Economic evaluation of the proposed butyric acid recovery process

Variable	Extractor	Dist. I	Dist. II	Dist. III
Investment				
Column	0.247	0.128	0.094	0.055
Tray	0.015	0.006	0.004	0.002
Reboiler		0.078	0.032	0.009
Condenser		0.069	0.061	0.022
Preheater/cooler	0.123	0.083	0.003	0.001
Subtotal	0.385	0.364	0.194	0.089
Total				1.032
Utility				
Steam		0.209	0.052	0.007
Coolant		0.002	0.002	0.001
Subtotal		0.211	0.054	0.008
Total				0.273

The units are in million U.S. dollars and the utility cost is given per annum

The comparison of the energy demand to the previously reported butyric acid/volatile fatty acids (VFAs) is presented in Table 4, and their investment costs are presented in Table 5. Although a poor feed composition consumes more recovery energy [4], the energy demand of this study is comparable to that of previous studies, except for a VFA recovery process designed with a less effective UNIFAC thermodynamic model [9]. The energy consumption of the proposed process is lower than the average energy demand of the other listed processes. This outcome was attributed to the judicious selection of the extraction solvent. The large amount of water in the feed significantly affects the energy demand in the case of biofuel processing.

**Table 4:** Energy demand comparison for volatile fatty acids (VFAs) recovery processes

Products	Solvent	Feedstock (wt%)	Reboiler duty (MW) for product t/h	Remark	Refs.
VFA	MTBE	5	3.35	Heat integrated	[53]
VFA	Ethyl acetate	5	2.69		[13]
Butyric acid	Octanol	5.5	5.89		[54]
VFA	Nonyl acetate/hexyl acetate	0.97	1.34		[9]
Butyric acid	Octyl acetate	2.67	2.72	Heat integrated	This study

The VFAs have two (acetic acid) to six (caproic acid) carbon atoms

**Table 5:** Investment cost comparison for various VFA recovery processes

Products	Capacity (product t/h)	Investment	Normalized	Remark	Refs.
VFA	6.2	16.2	1.92		[53]
VFA	22	65.1	3.27	Scaled	[13]
Butyric acid	1.25	13.5	4.76		[54]
VFA	0.1	1.26	2.02		[9]
Butyric acid	0.27	1.03	1.03		This study

The units are in million US dollars. The scaled investment is computed from total investment using the ratio of the purification process in [53, 54]. The investment normalization is based on this study used a scaling-up exponent of 0.68 [55]

The investment costs of various butyric acid/VFA recovery processes were compared, where the cost was shown in the original and normalized numbers by their production capacity. Although their production capacity varies widely, a normalization using a scaling-up exponent of 0.68 [55] would provide a crude comparison of the investment. The results of this study indicate a significant reduction in the investment cost of the recovery process.

### Possible improvements

Owing to the nature of fermentation, a significant amount of wastewater is generated from the process, and the same amount of water is consumed at the beginning of fermentation. Recycling the wastewater would address the problem of feed water supply and wastewater treatment. Numerous studies on solvent toxicity have been conducted to evaluate the feasibility of wastewater recycling [56–58]. The solvent used in this study, octyl acetate, is toxic to bacteria; therefore, it is necessary to remove it prior to recycling. Tridecanol is nontoxic to bacteria [56] and is a suitable solvent for removing octyl acetate. Table 6 summarizes the extraction results obtained using a 21-stage extractor for processing wastewater. The resultant low octyl acetate content (0.9 ppm) present in recycled wastewater would not harmful to microorganisms during butyric acid fermentation [59]. Acetic acid in the wastewater was also recovered via recycling.

**Table 6:** Stream flow rates and compositions for wastewater treatment with tridecanol extraction

Stream	Wastewater	Solvent	Raffinate	Extract
Composition				
Water	0.9948	0	0.9962	0.1283
Acetic acid	0.0037	0	0.0037	0.0028
Butyric acid	0.0002	0	0.0001	0.0010
Octyl acetate	0.0013	0	0 (0.9 ppm)	0.0692
Tridecanol	0	1	0	0.7987
Flow rates (kg/h)	9682	150	9644	187.6

## Conclusions

A butyric acid recovery process was proposed, wherein octyl acetate was used as the extraction solvent, and the process design, including that of extraction, was investigated in detail. The LLE tie-line data used for the extractor design were obtained from molecular simulations and experimental measurements. The procedure for the estimation of thermodynamic parameters used for the extractor design was improved by eliminating the need for iteration to solve the phase equation. The compatibility of the parameters with a commercial process design program was examined to ensure accuracy of the extractor design results, which determine the integrity of the entire recovery process. Butyric acid was recovered with a purity of 99.8% at a rate of 99% recovery. The energy demand of the proposed process is lower than the average demand of several butyric acid recovery processes. The investment cost was lower than that of the compared processes due to the high efficiency of extraction solvent. The recovery cost of $0.23/kg butyric acid was comparable to other process and its selling price of $2.70/kg.

## Supplementary Information

**Additional file 1: Table S1.** Binary interaction parameters in the NRTL models. **Table S2.** Measurements of the liquid–liquid equilibrium in the water(1)/butyric acid(2)/octyl acetate(3) system at T = 298.15 K and p = 101.3 kPa. Units are in mole fractions. The standard uncertainties are *u*(mole frac.) = 0.003, *u*(T) = 0.1 K, and *u*(P) = 2 kPa. *D* is the distribution coefficient of the solute and *S* is the solute selectivity in the organic to aqueous phases. **Table S3.** Calculated compositions of liquid–liquid equilibrium in a system of water(1)/butyric acid(2)/octyl acetate(3) using molecular simulation and NRTL prediction. Units are in mole fraction.

## Data Availability

All data generated or analyzed during this study are included in this published article and its additional file.

## Abbreviations

COSMO-RS: Conductor-like screening model for real solvents
LLE: Liquid–liquid equilibrium
NRTL: Non–random two–liquid
TraPPE: Transferable potentials for phase equilibria
UNIFAC: UNIQUAC functional-group activity coefficients
UNIQUAC: Universal quasichemical
VFA: Volatile fatty acid
VLE: Vapor–liquid equilibrium
