# Supplementary material for: Energy-efficient recovery of fermented butyric acid using octyl acetate extraction
Source: Biotechnol Biofuels Bioprod. 2022 May 6;15:46. doi: 10.1186/s13068-022-02146-6 (PMC9074251; doi:10.1186/s13068-022-02146-6)
Supplement: Supplementary file 1 — Additional file 1: Table S1. Binary interaction parameters in the NRTL models. Table S2. Measurements of the liquid–liquid equilibrium in the water(1)/butyric acid(2)/octyl acetate(3) system at T = 298.15 K and p = 101.3 kPa. Units are in mole fractions. The standard uncertainties are u(mole frac.) = 0.003, u(T) = 0.1 K, and u(P) = 2 kPa. D is the distribution coefficient of the solute and S is the solute selectivity in the organic to aqueous phases. Table S3. Calculated compositions of liquid–liquid equilibrium in a system of water(1)/butyric acid(2)/octyl acetate(3) using molecular simulation and NRTL prediction. Units are in mole fraction. [file 13068_2022_2146_MOESM1_ESM.docx]

Energy-efficient recovery of fermented butyric acid

using octyl acetate extraction

Hyeonwoo Oh, Seong Chan Lee, Hee Chul Woo^*^, and Young Han Kim^*^

Department of Chemical Engineering, Pukyong National University

Additional file 1

Table S1. Binary interaction parameters in the NRTL models.

| System | Component i | Component j | a_ij_ | a_ji_ | b_ij_ | b_ji_ | α |
| --- | --- | --- | --- | --- | --- | --- | --- |
| VLE | Water | Acetic acid | 0 | 0 | 318.6806 | 24.1223 | 0.3 |
|  | Water | Butyric acid | 2.3604 | -2.0043 | 186.3757 | 694.8912 | 0.3 |
|  | Water | Octyl acetate | 0 | 0 | 283.584 | 1039.287 | 0.3 |
|  | Acetic acid | Butyric acid | 3.2775 | -1.7272 | 0 | 0 | 0.3 |
|  | Acetic acid | Octyl acetate | 0 | 0 | -421.785 | 228.4522 | 0.3 |
|  | Butyric acid | Octyl acetate | 0 | 0 | 134.1707 | -119.178 | 0.3 |
| LLE | Water | Acetic acid | 0 | 0 | 488.6082 | 420.5704 | 0.3 |
|  | Water | Butyric acid | 0 | 0 | 1203.721 | -249.701 | 0.2 |
|  | Water | Octyl acetate | 0 | 0 | 2265.105 | 544.0045 | 0.2 |
|  | Acetic acid | Butyric acid | 0 | 0 | 925.9346 | 53.81608 | 0.3 |
|  | Acetic acid | Octyl acetate | 0 | 0 | 986.7274 | 348.269 | 0.3 |
|  | Butyric acid | Octyl acetate | 0 | 0 | -672.895 | 1703.957 | 0.3 |

Table S2. Measurements of the liquid–liquid equilibrium in the water(1)/butyric acid(2)/octyl acetate(3) system at T = 298.15 K and p = 101.3 kPa. Units are in mole fractions. The standard uncertainties are *u*(mole frac.) = 0.003, *u*(T) = 0.1 K, and *u*(P) = 2 kPa. *D* is the distribution coefficient of the solute and *S* is the solute selectivity in the organic to aqueous phases.

| Number | Organic phase | | | Aqueous phase | | |  |  |
| --- | --- | --- | --- | --- | --- | --- | --- | --- |
|  | *x*_1,1_ | *x*_2,1_ | *x*_3,1_ | *x*_1,2_ | *x*_2,2_ | *x*_3,2_ | *D* | *S* |
| 1-1 | 0.1300 | 0.1475 | 0.7226 | 0.9935 | 0.0064 | 0.0000 | 23.0 | 176.1 |
| 1-2 | 0.1279 | 0.1440 | 0.7281 | 0.9887 | 0.0076 | 0.0038 | 18.9 | 146.5 |
| 1-3 | 0.1279 | 0.1440 | 0.7281 | 0.9887 | 0.0076 | 0.0038 | 18.9 | 146.5 |
| 2-1 | 0.1494 | 0.1963 | 0.6543 | 0.9924 | 0.0076 | 0.0000 | 25.8 | 171.6 |
| 2-2 | 0.1453 | 0.2046 | 0.6501 | 0.9953 | 0.0045 | 0.0001 | 45.5 | 311.4 |
| 2-3 | 0.1413 | 0.2059 | 0.6528 | 0.9962 | 0.0038 | 0.0000 | 54.2 | 382.0 |
| 3-1 | 0.1838 | 0.2750 | 0.5412 | 0.9898 | 0.0102 | 0.0000 | 27.0 | 145.2 |
| 3-2 | 0.1816 | 0.2778 | 0.5407 | 0.9911 | 0.0031 | 0.0058 | 89.6 | 489.1 |
| 3-3 | 0.1782 | 0.2752 | 0.5469 | 0.9944 | 0.0056 | 0.0000 | 49.1 | 274.2 |
| 4-1 | 0.1793 | 0.3336 | 0.4871 | 0.9905 | 0.0094 | 0.0004 | 35.5 | 196.1 |
| 4-2 | 0.1771 | 0.3340 | 0.4889 | 0.9963 | 0.0026 | 0.0011 | 128.5 | 722.7 |
| 4-3 | 0.1743 | 0.3345 | 0.4912 | 0.9963 | 0.0037 | 0.0000 | 90.4 | 516.8 |

Table S3 Calculated compositions of liquid-liquid equilibrium in a system of water(1)/butyric acid(2)/octyl acetate(3) using molecular simulation and NRTL prediction. Units are in mole fraction.

| Procedure | | Organic phase | | | | Aqueous phase | | |
| --- | --- | --- | --- | --- | --- | --- | --- | --- |
|  | *x*_1,1_ | | *x*_2,1_ | *x*_3,1_ | *x*_1,2_ | | *x*_2,2_ | *x*_3,2_ |
| Molecular simulation | | | | | | | | |
|  | 0.2070 | | 0.3776 | 0.4154 | 0.9804 | | 0.0196 | 0.0000 |
|  | 0.1494 | | 0.2791 | 0.5820 | 1.0000 | | 0.0000 | 0.0000 |
|  | 0.1122 | | 0.2604 | 0.6274 | 1.0000 | | 0.0000 | 0.0000 |
|  | 0.1697 | | 0.3321 | 0.4981 | 0.9882 | | 0.0118 | 0.0000 |
|  | 0.1039 | | 0.1473 | 0.7488 | 0.9977 | | 0.0023 | 0.0000 |
|  | 0.1194 | | 0.1732 | 0.7074 | 0.9976 | | 0.0024 | 0.0000 |
|  | 0.0945 | | 0.2233 | 0.6823 | 0.9906 | | 0.0094 | 0.0000 |
|  | 0.1468 | | 0.3053 | 0.5479 | 0.9955 | | 0.0045 | 0.0000 |
|  | 0.1830 | | 0.3481 | 0.5055 | 0.9912 | | 0.0088 | 0.0000 |
|  | 0.0774 | | 0.0997 | 0.8229 | 0.9982 | | 0.0018 | 0.0000 |
| NRTL | | | | |  | |  |  |
|  | 0.2006 | | 0.3787 | 0.4207 | 0.9877 | | 0.0121 | 0.0002 |
|  | 0.1821 | | 0.3545 | 0.4634 | 0.9897 | | 0.0102 | 0.0002 |
|  | 0.1688 | | 0.3343 | 0.4969 | 0.9910 | | 0.0088 | 0.0002 |
|  | 0.1486 | | 0.2985 | 0.5530 | 0.9931 | | 0.0068 | 0.0002 |
|  | 0.1417 | | 0.2846 | 0.5737 | 0.9937 | | 0.0061 | 0.0002 |
|  | 0.1283 | | 0.2546 | 0.6171 | 0.9949 | | 0.0049 | 0.0002 |
|  | 0.1153 | | 0.2213 | 0.6634 | 0.9960 | | 0.0039 | 0.0002 |
|  | 0.1000 | | 0.1762 | 0.7238 | 0.9970 | | 0.0028 | 0.0002 |
|  | 0.0959 | | 0.1635 | 0.7406 | 0.9973 | | 0.0026 | 0.0002 |
|  | 0.0790 | | 0.1080 | 0.8131 | 0.9980 | | 0.0018 | 0.0002 |
